# Lack of patient education is risk factor of disease flare in patients with systemic lupus erythematosus in China

**DOI:** 10.1186/s12913-019-4206-y

**Published:** 2019-06-13

**Authors:** Le Zhang, Wei Luan, Shikai Geng, Shuang Ye, Xiaodong Wang, Liping Qian, Yang Ding, Ting Li, Anli Jiang

**Affiliations:** 1 Department of Pharmacy, South Campus, Renji Hospital, School of Medicine, Shanghai Jiao Tong University, Shanghai, China; 2 School of Nursing, Shanghai Second Military Medical University, Shanghai, China; 3 Department of Rheumatology, South Campus, Renji Hospital, School of Medicine, Shanghai Jiao Tong University, Shanghai, China; 4 Department of Mathematics, Applied Statistics, Shanghai Jiao tong University, Shanghai, China; 5 Department of Nursing, South Campus, Renji Hospital, School of Medicine, Shanghai Jiao Tong University, No.2000 Jiangyue RD, Shanghai, 201112 China

**Keywords:** Beliefs about medicines, Satisfaction with medicines information, Systemic lupus erythematosus disease activity index, Patient education and consulting

## Abstract

**Background:**

To explore the inadequacies of health service and its impact on clinical outcomes of patients with systemic lupus erythematosus (SLE) in China.

**Methods:**

A total of 210 SLE patients were randomly recruited between January 2017 and January 2018. Each patient received self-report questionnaires to assess medication adherence [Compliance Questionnaire for Rheumatology (CQR)], beliefs about medicines [Beliefs about Medicines Questionnaire (BMQ)] and satisfaction about medicine information [the Satisfaction with Information about Medicines Scale (SIMS)]. Associations between SLE disease activity index (SLEDAI-2 K) and observed factors were analyzed by multiple logistic regression.

**Results:**

Based on CQR, only 28.10% patients were adherent. The score of BMQ was 2.85 ± 5.42, and merely 32.38% patients were satisfied with the information about their prescribed medicines. Disease activity was associated with SIMS, EuroQol five-dimensions [EQ5D], Systemic Lupus International Collaborating Clinics (SLICC), depression, use of NSAID (*P* ≤ 0.05). Remission of disease was positively correlated with SIMS (OR = 0.16, 95% CI: [0.06, 0.40]), and BMQ (OR = 0.64, 95%CI: [0.43, 0.94]).

**Conclusion:**

In this study, the scores of BMQ and SIMS were low, implying defects in the patient education of health service system, which led to disease flare in Chinese SLE patients.

**Trial registration:**

ClinicalTrials.gov ID: NCT03024307. Registered January 18, 2017.

**Electronic supplementary material:**

The online version of this article (10.1186/s12913-019-4206-y) contains supplementary material, which is available to authorized users.

## Background

Systemic lupus erythematosus (SLE) is an autoimmune disease characterized by a variety of autoantibodies in the blood and multiple system and organ involvements (skin, joints, lungs, heart, kidneys, brain, etc.) [1]. At present, although there is no cure for SLE, drugs treatment can prevent organ damage to the minimum and control disease active, which was based on glucocorticoids and immunosuppressants [2, 3]. Therefore, effective disease management on adherence to medicine is the key to ensure the treatment effect in SLE patients.

Previous studies of patients with SLE, which had different populations and methodologies, reported that adherence ranged from 3 to 76%, 67% for glucocorticoids, 48.6%~ 93% for hydroxychloroquine, and 57% for other immunosuppressants [4–6]. Besides, the rate of treatment adherence in China was about 48.7% based on our previous study [7]. Moreover, chronic rheumatic conditions are inclined to result in substantial burdens such as economic burden for patients and their families. In Europe, the per capita annual direct medical cost of patients with SLE reaches €4748 (US$5037.15), up to US$6269 [8–10] in the US. Surprisingly, the cost in our previous study of SLE population also reached $5103 in China [7], which is a huge expenditure for a developing country. The results of our previous study suggest that we have defects in the management of lupus patients, and we should take measures to improve their adherence and reduce the disease burden. Therefore, in order to explore specific problems in disease management of lupus patients in China, we carried out this research and hope to further find out the deficiency in the health service.

## Methods

### Study design

This cross-sectional study was conducted from 2017 to 2018 at the Renji Hospital, Shanghai, China. The research protocol was approved by Shanghai Jiao Tong University, School of Medicine, Renji Hospital Ethics Committee (approval No.[2016]216 K). This center is one of the largest rheumatology centers in China, and the patients are from all over the country. All participating patients provided written informed consent and completed questionnaires which assessed adherence to treatment, beliefs about medicines, and satisfaction with information on treatment drugs.

### Recruitment and data collection

Outpatients visiting the South Campus of Renji Hospital between January 2017 and January 2018 were considered for inclusion if they met the American College of Rheumatology (ACR) 2012 criteria for SLE and were being treated with rheumatic drugs.

Those illiterate, mentally disordered or with serious physical constraints were excluded. All other patients were included, regardless of their demographics, disease characteristics, or treatment characteristics. Data collected included the patients’ demographic characteristics (age, gender, marital status, education level, employment), disease characteristics (disease duration, comorbidities, and health status based on EuroQol five dimensions [EQ-5D] score, disease activity evaluated by SLEDAI-2 K [systemic lupus erythematosus disease activity index] [11, 12] and SLICC [Systemic Lupus International Collaborating Clinics] [13, 14]), and treatment characteristics (types of pills taken daily, use of a glucocorticoid (GC), use of immunomodulators and immunosuppressants [e.g. hydroxychloroquine, azathioprine, tacrolimus, etc.], use of non-steroidal anti-inflammatory drugs (NSAIDs), use of a biological drugs [e.g. Rituximab, etc.], daily dosing frequency, and side effects).

Disease activity was evaluated at baseline by the SLEDAI-2 K instrument. It is a valid, reliable and widely used approach to measure disease activity in SLE patients. The scores range from 0 to 105. Medication adjustment is considered necessary if the score difference between two successive evaluations of a patient is greater than 6. The patients were divided into four groups: inactive (0–4 points), mild (5–9 points), moderate (10–14 points), or severe disease activity (≥15 points). Forty-one items of target organ damages were also assessed using the SLICC-index.

### Self-reported adherence

#### CQR assessment

Self-reported adherence was assessed using Compliance Questionnaire for Rheumatology (CQR). It was proved of good reliability and validity in lupus patients by previous study [15]. The CQR consists of 19 items about taking medicine, in which patients were asked the degree of agreement with statements. Answers are based on four-point Likert scales from 4 to 1 [16] (4: agree very much;3: agree; 2: do not agree; 1: do not agree at all;). The final point allows the identification of non-adherent patients (defined as poor compliance ≤80%) with a small false-positive rate.

#### Beliefs about medicines

Patient beliefs about medicines were assessed using the Beliefs about Medicines Questionnaire, BMQ. This questionnaire has been confirmed of its reliability and validity before [17], and we also found Cronbach’s α of 0.88 for necessity scale and 0.77 for concern scale in this study (Additional file 1). The BMQ-specific quantifies patients’ beliefs about the necessity of a prescribed medication to control their disease, as well as their concerns about the potential side effects of the medication [18]. Both beliefs about necessity and concerns about side effects were measured in terms of 5 items rated on a 5-point Likert scale and the total scores of the Necessity and Concerns scales range from 5 to 25. By subtracting a patient’s concerns score from the his/her necessity score, a necessity–concerns differential was obtained, ranging from − 20 to 20 [19]. Higher differential scores indicate the stronger perceived necessity about medication necessity and/or lower concerns about its side effects.

#### Satisfaction with information on treatment

Levels of patients’ satisfaction with information on treatment were evaluated using the Chinese version of the validated Satisfaction with Information about Medicines Scale (SIMS). It investigates whether patients feel that they have been given adequately informed on prescribed drugs [20]. The questionnaire comprises 18 items, each relating to a particular aspect of drug use. An overall satisfaction rating was obtained by summing the scores of all 18 items, ranging from 0 to 18 [21]. The higher the score, the greater satisfaction a patient felt with information. Patients were asked to make satisfaction ratings on the information provided for them using the 5 items: “too much”, “about right”, “too little”, “none received”, and “none needed”. Ratings of “about right” or “none needed” suggested patients’ satisfaction with the information and were given a score of 1, while ratings of “too much”, “too little” or “none received” suggested dissatisfaction and were scored as 0. The internal reliability of the scale scores was good with a Cronbach’s α coefficient of 0.95. Total score of items 1–9 was used to measure patients’ satisfaction with information on action and usage (score ranges from 0 to 9) (Cronbach’s α 0.94); total score of items 10–18 was used to measure the satisfaction with information on potential problems (score ranges from 0 to 9) (Cronbach’s α 0.94) (Additional file 1). For the three SIMS scales, median scores were used to define dissatisfaction (< 16 of 18 items for overall satisfaction rating; < 8 of 9 items for subscale of action and usage and potential problems). In consideration of the left-skewed distribution of the three scales, they were dichotomized into satisfaction coded as 1 versus dissatisfaction as 0.

#### Measurement of health status

The quality of life was accessed using the Chinese version of the general population-based three-level EuroQol five-dimensions questionnaire [EQ-5D-3 L] [22, 23]. Each EQ-5D-3 L health state was scored as 1 (no problems), 2 (some/moderate problems), or 3 (extreme problems) to indicate functional levels in five dimensions: mobility, self-care, usual activities, pain/discomfort, and anxiety/depression. The EQ-5D-index was calculated by “time-trade-off”, to assess the patients’ quality of life.

#### Statistical analysis

Descriptive statistics were used to analyze the demographics and patient characteristics. The categorical data were summarized as numbers and percentages, while the continuous data were summarized as the mean and standard deviation. According to the SLEDAI-2 K, all SLE patients were divided into two groups: the Minimal-Mild group (SLEDAI-2 K ≤ 9) and the Moderate-Severe group (SLEDAI-2 K > 10). A Chi-square test was used to compare the categorical variables of the two groups, whereas the Student t-test was used for continuous data comparison. Data fitting was conducted by using the multinomial logistic regression analysis model, and the influence of each feature on the target value was determined. Variables (*P* ≤ 0.05) were included in logistic regression analyses so that the strength of the multivariate association could be quantified, and BMQ was also included as a key factor. Then the best model was obtained through a stepwise logistic regression analysis. Odds ratios with 95% confidence intervals were used. All analyses were performed by R software (version 3.4.2).

## Results

### Study sample

The data of demographic and clinical characteristics of the study population (*n* = 210) was shown in Table 1. A total of 191 patients (90.95%) were female and the overall mean (±SD) age was 36.44 (±12.83) years. A total of 70.48% of the patients were married, 50% were employed, and 50% had a high education (>12 years).

**Table 1:** Demographic characteristics and clinical manifestations of the patient population (*n* = 210)

Sociodemographic characteristics	Descriptive
Age (years), mean (SD)	36.44 (12.83)
Sex, female, *n* (%)	191 (90.95)
Marital status, *n* (%)	
Married	148 (70.48)
Other marital status	62 (29.52)
Education level, *n* (%)	
Primary (0–6 years)	27 (12.86)
Secondary (7–12 years)	78 (37.14)
Higher (>12 years)	105 (50.00)
Employment, *n* (%)	
Employed	105 (50.00)
Unemployed	105 (50.00)
Disease characteristic	Descriptive
Disease duration, *n* (%)	
<1 year	12 (5.71)
1–5 year	98 (46.67)
≥ 5 year	100 (47.62)
Comorbidities, *n* (%)	
0	75 (35.71)
1	75 (35.71)
2	33 (15.71)
≥ 3	27 (12.86)
EQ-5D Index, mean (SD)	0.72 (0.26)
SLEDAI-2 K	
0–4	98 (46.67)
5–9	50 (23.81)
10–14	41 (19.52)
≥ 15	21 (10.00)
SLICC Index, mean (SD)	0.87(1.26)
Treatment characteristic	Descriptive
Types of pills prescribed daily, mean (SD)	4.86 (2.20)
Use of GC, *n* (%)	198 (94.29)
Number of immunomodulators&immunosuppressants, mean (SD)	0.89 (0.70)
Use of NSAID, *n* (%)	18 (8.57)
Use of biologic drugs, *n* (%)	22 (10.47)
Daily dosing frequency, *n* (%)	
< once daily	27 (12.86)
once daily	46 (21.90)
Twice daily	87 (41.43)
Thrice daily	47 (22.38)
> Thrice daily	3 (1.43)
Side effects, *n* (%)	
0	175(83.33)
1–2	28(13.33)
≥ 3	7 (3.33)
CQR19, mean (SD)	74.06 (8.93)

*Abbreviations*: *CQR* compliance questionnaire rheumatology, *EQ-5D* EuroQol five-dimensions, *GC* glucocorticoid, *NSAID* non-steroidal anti-inflammatory drug, *SD* standard deviation, *SLEDAI* systemic lupus erythematosus disease activity index, *SLICC* the Systemic Lupus International Collaborating Clinics

Analysis of disease characteristics showed that 68 patients (47.62%) had disease durations over 5 years. Most patients had 1~2 comorbidities (51.43%). The overall mean EQ-5D index was 0.72 (± 0.26). In our patients, the clinical SLEDAI-2 K ≤ 4 points contains 98 individuals (46.67%), 50(23.81%)between 5 and 9 points. 41 (19.52%) patients scored 10 to 14 and 21 (10%) were more than 15 points which implied intense disease activity. One hundred and-thirteen patients (53.81%) showed no organ damages with 0 point according to SLICC.

Analysis of treatments indicated that these patients took an average of 4.86 (±2.20) types of drugs daily. The most commonly used drugs were GCs (94.29%), NSAIDs (8.57%), and biological drugs (10.47%). These patients also received an average of 0.89 (± 0.70) different kinds of immunomodulators and immunosuppressants, and 41.43% of patients took medicines twice daily. The mean CQR score was 74.06 (± 8.93) (Table 1).

### Internal consistency

Cronbach’s alpha coefficients for the CQR, BMQ-necessity, BMQ-concern, and SIMS- action and usage, SMIS-potential problems, SIMS obtained in each sample were 0.87, 0.88, 0.77, 0.94, 0.94, 0.95. The complete CQR, BMQ, and SIMS showed good internal reliability in this study (Additional file 1).

### Self-reported adherence

According to the results of CQR, 28.10% were adherent.

### Beliefs about medicines questionnaire-specific

The average levels of necessity beliefs were high (mean score 19.33 ± 3.66). The mean concern score for BMQ-concern was 16.48 (3.76). The score of BMQ on average in all patients with SLE was 2.85 (5.42), which indicated lupus patients had more concerns about the treatment drugs than necessity. In patients with minimal-mild disease activity, they had a high total score of BMQ, illustrating that they had better beliefs about medicine treatment (Table 2).

**Table 2:** Beliefs about necessity and concerns between minimal-mild and moderate-severe patients (*n* = 210)

Necessity Scale	*n* (%) agreeing or strongly agreeing		Concern Scale	*n* (%) agreeing or strongly agreeing	
	Minimal-Mild *n* = 148	Moderate-Severe *n* = 62		Minimal-Mild *n* = 148	Moderate-Severe *n* = 62
At present, my health depends on medication	113 (76.35)	52 (83.87)	Having to take medicines worries me	86 (58.11)	42 (67.74)
My life would be impossible without medication	93 (62.84)	47 (75.81)	I sometimes worry about the long-term effects of my medicines	110(74.32)	57 (91.94)
Without medication I become very ill	120 (81.08)	53 (85.48)	My medicines disrupt my life	47 (31.76)	36 (58.06)
My future health depends on medication	113 (76.35)	52 (83.87)	My medicines disrupt my life	51 (34.46)	26 (41.94)
Medication protects me	133 (79.12)	57 (91.94)	I sometimes worry about becoming too dependent on my medicines	85 (67.43)	48 (77.42)
Score of BMQ-Necessity, mean (SD)	19.23 (3.73)	19.56 (3.50)	Score of BMQ-Concern, mean (SD)	16.01 (3.86)	17.60 (3.29)
Score of BMQ, mean (SD)	3.21 (5.57)	1.97 (4.96)			

*Abbreviations*: *BMQ* beliefs about medicines questionnaire, *SIMS* the satisfaction with information about medicines scale

### Satisfaction with information on treatment

Among all patients, only 32.38% lupus patients were satisfied with the information about their prescribed medicines. In the two subscales, 46.67% patients were satisfied with the information about drug action and usage, and 36.67% were satisfied with the information about drug potential problems (Table 3)**.** Compared with patients with moderate-severe disease activity, patients with minimal-mild had a better SIMS score, including SIMS- action and usage and SIMS-potential problems. From the overall results, it implied our shortcomings in our health service, lacking in medication education for patients.

**Table 3:** Patient satisfaction with information on treatment in minimal-mild and moderate-severe patients (Satisfaction with Information about Medicines Scale [SIMS]) (*n* = 210)

SIMS- action and usage	n (%) Satisfied		SIMS-potential problems	*n* (%) Satisfied	
	Minimal-Mild *n* = 148	Moderate-Severe *n* = 62		Minimal-Mild *n* = 148	Moderate-Severe *n* = 62
Medicine name	115(77.70)	32(51.61)	Which side effects	96(64.86)	23(37.10)
Indication	106(71.62)	33(53.23)	Side effect risk	86(58.11)	22(35.48)
Effects	105(70.95)	34(54.84)	What to do when side effects occur	87(58.78)	17(27.42)
Mechanism	93(62.84)	25(40.32)	Interactions	84(56.76)	16(25.81)
Duration effects	88(59.46)	22(35.48)	Alcohol use	100 (67.57)	22(35.48)
Perceived effects	91 (61.49)	23(37.10)	Drowsiness	92(62.16)	15(24.19)
Duration medication use	96(64.86)	27(43.55)	Effect on sex life	97 (65.54)	17(27.42)
Usage	113(76.35)	36(58.06)	Missed doses	86(58.11)	16(25.81)
Followup prescriptions	101 (68.24)	30(48.39)	Effects on the unborn child	98(66.22)	24(38.71)
Total proportion of SIMS- action and usage	80(54.05)	18(29.03)	Total proportion of SIMS-potential problems	67 (45.27)	10(16.13)
Total proportion of SIMS	58(39.19)	10(16.13)			

*Abbreviations*: *SIMS* the satisfaction with information about medicines scale

### Measurement of health status

The overall mean EQ-5D index was 0.72 (± 0.26). In the 5 dimensions, the number of patients who have problems in anxiety/depression is the most (92, 43.81%), 87 patients having some problems for anxiety/depression, 5 patients having extreme problems for it. 41.43% patients had problem in pain/discomfort and usual activities, 32.38% having problems for mobility, 22.86% having problems for self-care. As the highest proportion of problems, anxiety/depression was also included in Univariate analysis of factors associated with disease activity.

### Univariate analysis of factors associated with disease activity

**Fig. 1:**
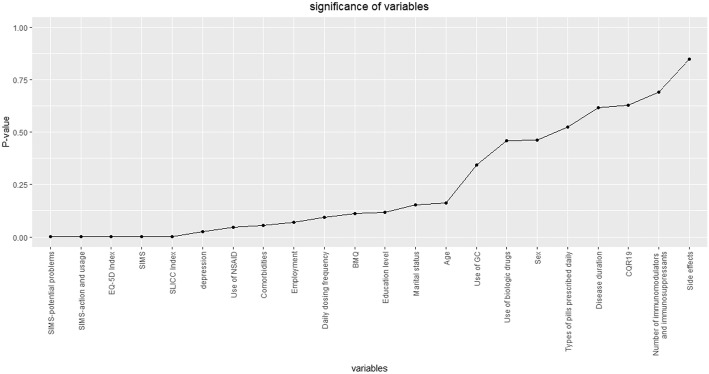
Significance of demographic and clinical characteristics to SLEDAI. *P < 0.05. **P < 0.01. ***P < 0.001

The results of univariate analysis of the association between different demographic and clinical characteristics and disease activity were showed in Fig. 1**.** Among all 210 patients, disease activity was associated with SIMS, EQ-5D, anxiety/depression, SLICC, use of NSAID (*P* ≤ 0.05 for all comparisons). Table with exact *p*-values could be found in Additional file 2.

### Multiple logistic regression analysis of factors associated with disease activity

In order to clarify the specific impact trend of above factors on disease activity, a multiple logistic regression analysis was used. According to the logistic regression diagnostics, noisy data, outliers, high-leverage points and influential observations were identified and then exclude from the logistic regression (Additional file 2). Of all 204 patients, minimal-mild disease activity was associated with high scores of SIMS (OR = 0.16, 95%CI: [0.06, 0.40], *P* < 0001) and BMQ (OR = 0.64, 95%CI: [0.43, 0.94], *P* < 0.05). While moderate-severe disease activity was associated with high scores of SLICC (OR = 2.29, 95%CI: [1.54, 3.51], *P* < 0.0001), and use of NSAID (OR = 3.64, 95%CI: [1.11, 12.34], *P* < 0.05), implying good reliability of the analytical model (Table 4).

**Table 4:** Logistic regression for the predictors of disease activity among patients with SLE (*n* = 204)

Variable	Level	Odds ratio	95% CI low	95% CI high	*p*-value
SLICC Index	One-unit increase	2.29	1.54	3.51	0.0001
SIMS	satisfied	0.16	0.06	0.40	0.0002
BMQ	One-unit increase	0.64	0.43	0.94	0.0297
NSAID	Yes	3.64	1.11	12.34	0.0332

*Abbreviations*: *BMQ* beliefs about medicines questionnaire, *NSAID* non-steroidal anti-inflammatory drug, *SLICC* the Systemic Lupus International Collaborating Clinics, *SIMS* the satisfaction with information about medicines scale

## Discussion

To the best of our knowledge, this is the first study to prove the importance of health service on clinical outcomes in SLE patients. In this study, we use self-report questionnaires to assess adherence and examine the medication belief and satisfaction with drug information in patients with SLE in mainland China. The level of self-reported adherence (28.10%) and satisfaction rate (32.38%) are low among our patients. Besides, it had a negative effect on the clinical outcomes, according to multiple logistic regression analysis. It suggested that we neglect patient education in medical services in our country.

Similar to previous study [24–28], we also found that disease activity in patients with SLE was associated with EQ5D, anxiety/depression, SLICC, use of NSAID. Through further analysis, we found disease activity in SLE patients was associated with BMQ and SIMS, which indicated that the medication belief and satisfaction with drug information may affect the disease activity [29]. It is proved by our study that poor medication education had a negative impact on the clinical outcomes of SLE patients. Poor patient education is a barrier to successful treatment and is a challenge to health-care professionals [30].

Previous study on patients with SLE found that the score of BMQ-necessity ranged from 19.3 to 20.1, and the score of BMQ-concern ranged from 14.2 to 18.0 [31]. In our study, we found the score of BMQ-necessity was 19.33 ± 3.66, and BMQ-concern was 16.48 ± 3.76. It indicated our patients had more concerns about side effects than necessity about the treatment drugs. What’s more, we also found increasing scores of BMQ (OR = 0.64, 95% CI: [0.43, 0.94], *P* = 0.0297) were associated with minimal-mild disease activity, illustrating that good beliefs about medicine treatment was associated with remission of disease. The reason of this finding might be that stronger medication beliefs could lead to better treatment adherence [32, 33].

In the survey of satisfaction with medication information, only 32.38% were satisfied with the information about their prescribed medicines. In the two subscales, 46.67% patients were satisfied with the information about drug action and usage, and 36.67% were satisfied with the information about drug potential problems. It exposed our shortcomings in our work, lack of medication education for patients, especially about potential problems. This could be the reason why our patients had lots of concern about drugs, leading to anxiety/depression in the EQ5D, which was confirmed by previous study [20, 34, 35]. Besides, from the results of logistic regression analysis, increasing scores of SIMS (OR = 16, 95% CI: [0.06, 0.40], *P* = 0.0002) were associated with minimal-mild disease activity. Therefore, effective disease management, including information education of drugs, is the key to ensure patients to execute the treatment plan strictly and remission of disease [36].

There were some limitations in our work. First, there was a small number of patients, which was partly due to rarity of systemic lupus erythematosus. Second, our patients may not be totally representative of Chinese patients in general. However, our hospital is the largest rheumatology center in China, and our patients are from all over the country, so our research results are valuable. Further research on this topic should seek to enroll more patients from different area. Nevertheless, our results demonstrate that medication education potentially affect disease activity (the SLEDAI-2 K). As a chronic disease, the treatment compliance of patients with lupus plays a very important role in the clinical outcome. In the past, many studies aimed at improving patient compliance failed to achieve the desired results, because of switching among the drugs [37], patients’ lack of knowledge about disease and drugs [38], racial composition and hospital concentration [39]. Thus, an interventional study is needed to identify other measures which could improve clinical outcomes in the future.

## Conclusion

The findings of the study implied our shortcomings in the medical service among SLE patients and failure of medicine education for patients in China, leading to the poor scores of BMQ and SIMS and follow-up high disease activity. The results suggested that we should improve patient education to make them obtain sufficient information about drugs to promote their understanding about adverse drug reactions and to strengthen their belief in medication.

It was proved by the study that poor patient education in China had a negative impact on clinical outcomes. As the one of the largest rheumatology centers in China, we have established a multidisciplinary team, including pharmacists for professional education and consulting. We hope to increase patient satisfaction, medication belief and compliance through multidisciplinary management. Further intervention experiments is necessary to find measures to improve patient education in Chinese lupus patients.

## Additional files

Additional file 1: Internal consistency (Cronbach’s alpha) of the questionnaires. (DOCX 16 kb)

Additional file 2: Logistic Regression diagnostics. (DOCX 29 kb)

## Data Availability

Please contact the author for data requests: Zhang Le (joyce66dbl@hotmail.com).

## Abbreviations

ACR: American College of Rheumatology
BMQ: Beliefs about Medicines Questionnaire
CQR: Compliance Questionnaire for Rheumatology
EQ5D: EuroQol five-dimensions
GC: glucocorticoid
NSAIDs: non-steroidal anti-inflammatory drugs
SIMS: Satisfaction with Information about Medicines Scale
SLE: Systemic lupus erythematosus
SLEDAI: SLE disease activity index
SLICC: Systemic Lupus International Collaborating Clinics
