# Supplementary material for: Lack of patient education is risk factor of disease flare in patients with systemic lupus erythematosus in China
Source: BMC Health Serv Res. 2019 Jun 13;19:378. doi: 10.1186/s12913-019-4206-y (PMC6567412; doi:10.1186/s12913-019-4206-y)
Supplement: Supplementary file 1 — Internal consistency (Cronbach’s alpha) of the questionnaires. (DOCX 16 kb) [file 12913_2019_4206_MOESM1_ESM.docx]

**Additional file 1**

Internal consistency (Cronbach’s alpha) of the questionnaires

| Scales of the questionnaire | Range of score | Cronbach's α | Scoring above the scale midpoint |
| --- | --- | --- | --- |
| CQR | 19 TO 76 | 0.87 | 94.76% |
| BMQ-necessity | 5 TO 25 | 0.88 | 84.29% |
| BMQ-concern | 5 TO 25 | 0.77 | 63.33% |
| BMQ-specific (Necessity–Concerns) | -20 TO 20 | / | 64.76% |
| SIMS-action and usage | 0 TO 9 | 0.94 | 61.43% |
| SMIS-potential problems | 0 TO 9 | 0.94 | 49.05% |
| SIMS | 0 TO 18 | 0.95 | 52.86% |

**Abbreviations:** BMQ, the Beliefs about Medicines Questionnaire. CQR, compliance questionnaire rheumatology. SIMS, Satisfaction with Information about Medicines Scale.
