# Supplementary material for: Lack of patient education is risk factor of disease flare in patients with systemic lupus erythematosus in China
Source: BMC Health Serv Res. 2019 Jun 13;19:378. doi: 10.1186/s12913-019-4206-y (PMC6567412; doi:10.1186/s12913-019-4206-y)
Supplement: Supplementary file 2 — Logistic Regression diagnostics. (DOCX 29 kb) [file 12913_2019_4206_MOESM2_ESM.docx]

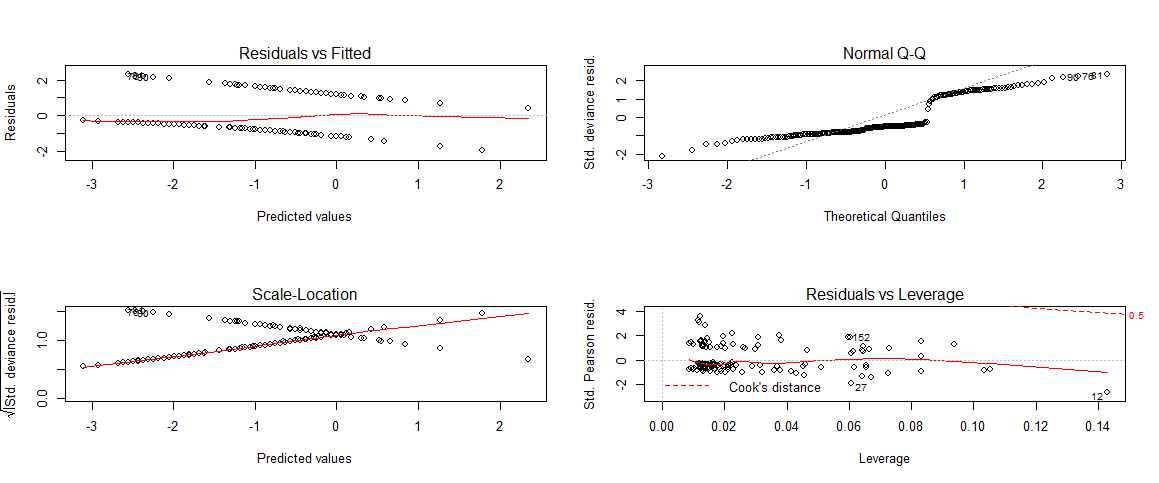


**Additional file 2**

Logistic Regression diagnostics

Noisy data, outliers, high-leverage points and influential observations were identified and then exclude from the logistic regression analysis.
